# Non-contact visual control of personalized hand prostheses/exoskeletons by tracking using augmented reality glasses

**DOI:** 10.1186/s41205-020-00059-4

**Published:** 2020-02-24

**Authors:** Simon Hazubski, Harald Hoppe, Andreas Otte

**Affiliations:** 1 Laboratory of Computer Assisted Medicine, Offenburg University, Badstr. 24, D-77652 Offenburg, Germany; 2 Laboratory of NeuroScience, Division of Medical Engineering, Department of Electrical Engineering, Medical Engineering and Computer Science, Offenburg University, Badstr. 24, D-77652 Offenburg, Germany

**Keywords:** Personalized prostheses, 3D-print, Visual control, Augmented reality

## Abstract

A new concept for robust non-invasive optical activation of motorized hand prostheses by simple and non-contact commands is presented. In addition, a novel approach for aiding hand amputees is shown, outlining significant progress in thinking worth testing. In this, personalized 3D-printed artificial flexible hands are combined with commercially available motorized exoskeletons, as they are used e.g. in tetraplegics.

Current approaches to controlling electrical neuroprostheses are based on measuring electromyography (EMG) signals of still existing muscles or using brain-machine or nerve-machine interface concepts to evaluate neuronal patterns and derive commands for the prosthesis from brain arrays, intrafascicular nerve electrodes, or combined electroencephalography/electrooculography (EEG/EOG) devices [1]. These neuroprosthetic concepts are intriguing and developing rapidly, albeit some of them are invasive or discomforting for the user and may not always reflect his or her wishes seeking for a smart but as simple as possible prosthesis, which can be attached, used, and controlled independently [2]. Some encouraging non-invasive and low-cost approaches have been developed, but most of them still require extended support, e.g. when electrodes of a non-invasive EEG/EOG system have to be attached.

In our new concept (Fig. 1), the only interface to the patient is with augmented reality (AR) technology by optical see-through glasses (OSTG), equipped with a front camera. The hand prosthesis may be any active motorized hand prosthesis or robotic arm. Markers are attached on the prosthetic hand, which may be (infrared) light emitting diodes (LEDs) or glued points. If the camera recognizes the markers, the transformation from the coordinate system of the hand prosthesis to the coordinate system of the AR glasses can be determined by means of an already developed algorithm [3]. As a result, the condition is created to evaluate the viewing direction of the viewer. The direction of view is the orientation of the glasses to the hand prosthesis, not the direction of the eyes. On or near the prosthesis is a virtual control panel. This control panel can either be permanently superimposed by the AR glasses or only be superimposed when the viewing direction approaches this region. In the simplest case, the control panel could contain virtual “push buttons” that trigger when the viewer’s gaze persists for more than one second, for example. In addition, the user could also execute commands in the form of defined minimal movements of the viewing direction.

**Fig. 1:**
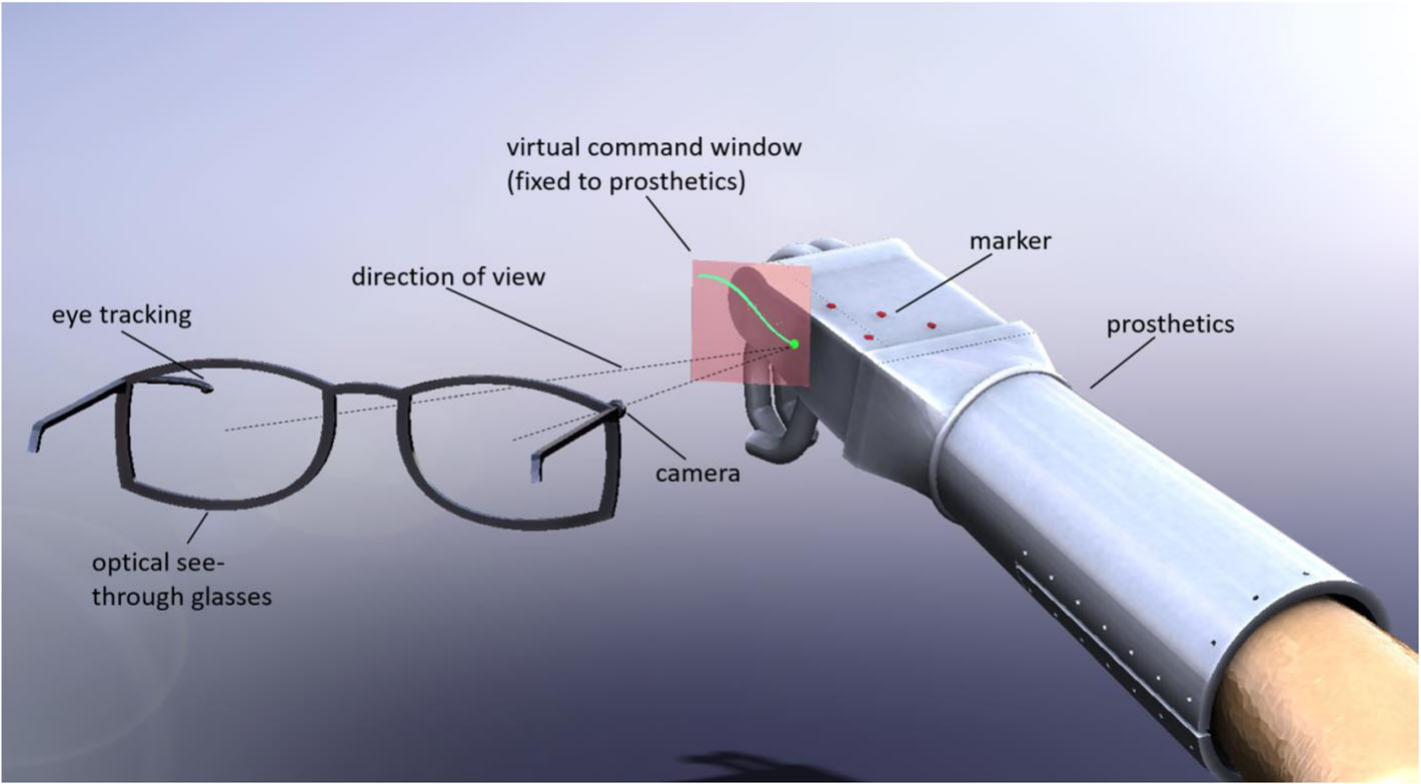
Schematic representation of the visual control for the hand prosthesis

The evaluation of the commands can be efficiently implemented by means of an artificial neural network. Among others, following commands are conceivable:

1. Viewing direction of the observer goes through command field from upper left corner to lower right corner → Prosthesis closes hand completely
2. Movement in the opposite direction as under 1. → Prosthesis opens hand completely
3. Viewing direction of the observer passes through command field from upper left corner to lower right corner only partially → Prosthesis closes hand in half
4. Partial movement in the opposite direction as in 3. → Prosthesis opens hand in half
5. Circular movement of the line of vision → Thumb moves

The commands can be customized according to the functions of the prosthetic hand and the preferences of the user. For instance, in hand prostheses which automatically switch off at a certain contact pressure, only the on/off command suffices. The user receives feedback, for example, by a trace of his/her movements of the viewing direction on the control panel or by a virtually displayed control panel. Optionally, the OSTG can be equipped with a camera for eye gaze tracking. The additional use of eye tracking can improve the accuracy of the recognition of the commands in the virtual control panel, as the user’s current viewing direction can be determined from the eye position, which may be important for certain neurological indications such as severe traumatic brain injury, stroke, amyotrophic lateral sclerosis, or other conditions, in which head movement is limited or completely impossible.

Non-invasive fully-independent approaches to aid amputees or tetraplegics outside of the laboratory environment are on its way [4]. Non-contact visual control of neuroprostheses by AR glasses could be an alternative to conventional EMG- or EEG/EOG-driven concepts worth looking for. Up to now, AR glasses have only been included into current EMG or EEG/EOG intention detection methods as an add-on to the concept [5, 6]. Solely using AR glasses–without any other complex signal detection by EMG, EOG or EEG–to control the prosthesis or robot hand is new and may simplify usability of prosthetic devices for the patient in the near future.

Low-cost 3D-printed motorized hands, controlled by OSTG and driven by energy-efficient neural network platforms could have an impact. Another step towards personalizing hand prostheses in amputees could be to use an easy-to-handle and inexpensive standard motorized exoskeleton instead of a sophisticated motorized hand prosthesis and to replace the missing hand with a LASER scan of the healthy hand (if available) or a volunteer’s hand. If a healthy hand can be scanned, the data could be mirrored to the other side of the person to replace the missing hand and 3D printed using flexible and lightweight polymer material. This personalized hand replacement could then be equipped with the exoskeleton in the same way as e.g. a tetraplegic’s hand.

## Data Availability

Not applicable.
